# Reticular Basement Membrane Vessels Are Increased in COPD Bronchial Mucosa by Both Factor VIII and Collagen IV Immunostaining and Are Hyperpermeable

**DOI:** 10.1155/2012/958383

**Published:** 2012-02-27

**Authors:** Amir Soltani, Richard Wood-Baker, Sukhwinder S. Sohal, H. Konrad Muller, David Reid, E. Haydn Walters

**Affiliations:** Menzies Research Institute, University of Tasmania, Private Bag 23, Hobart, TAS 7000, Australia

## Abstract

*Background and Objective*. Using Collagen IV staining, we have previously reported that the reticular basement membrane (Rbm) is hypervascular and the lamina propria (LP) is hypovascular in COPD airways. This study compared Collagen IV staining with vessels marked with anti-Factor VIII and examined vessel permeability in bronchial biopsies from COPD and normal subjects using albumin staining. *Results*. Anti-Collagen IV antibody detected more vessels in the Rbm (*P* = 0.002) and larger vessels in both Rbm (*P* < 0.001) and LP (*P* = 0.003) compared to Factor VIII. COPD airways had more vessels (with greater permeability) in the Rbm (*P* = 0.01) and fewer vessels (with normal permeability) in the LP compared to controls with both Collagen IV and Factor VIII antibodies (*P* = 0.04 and *P* = 0.01). *Conclusion*. Rbm vessels were increased in number and were hyperpermeable in COPD airways. Anti-Collagen IV and anti-Factor VIII antibodies did not uniformly detect the same vessel populations; the first is likely to reflect larger and older vessels with the latter reflecting smaller, younger vessels.

## 1. Introduction

Angiogenesis is under vigorous study in many diseases including chronic inflammation and malignancies. Chronic inflammatory diseases of the airways such as asthma and COPD are no exceptions [1, 2].

For better detection of blood vessels, specific stains are needed as haematoxylin and eosin alone are not specific enough [3]. The most commonly used tissue vessel markers in studies of the respiratory tract have been antibodies against Collagen IV and Factor VIII, CD31 (EN-4) and CD34. Both glycol methacrylate (GMA) processing and paraffin embedding are superior to other methods for investigation of vessels in tissue samples [4].

Factor VIII antigen is produced by endothelial cells and is physiologically involved in platelet aggregation and adhesion [5, 6]. A number of studies have reported Factor VIII antibody as a reliable marker for blood vessel detection [7]. However, it has been reported that Factor VIII antibody also stains megakaryocytes, mesenchymal tissue, and immune cells in addition to endothelial cells [4, 8]. The efficiency of Factor VIII antibody for detecting blood vessels has also been shown to be related to the size of vessels [9, 10].

Our group has had substantial experience in using Collagen IV antibody as a blood vessel marker in bronchial biopsies (BB) [2, 11–14]. Collagen IV antibody delineates endothelial basement membrane [3, 4].

An optimal marker for blood vessels should be specific, independent of pathological changes in tissue (e.g., inflammation, malignancy, hypoxia, ischemia, shearing stress), resistant to the usual methods of tissue fixation and processing, open to detection of a variety of sizes (i.e., large and small) and ages (i.e., old and new) of vessels, and be able to detect different types of vessels, that is, capillary, vein, arteriole, and artery. It has been shown that under both physiological and pathological conditions, endothelial cells modify their antigen presentation [10], and most available histochemical markers do not fully meet these characteristics, but the pros and cons of different immunohistologic antibody systems have not been worked out in any detail.

Antibodies to Collagen IV and Factor VIII stain different epitopes and indeed different structures in vessels, and the literature indicates that these markers for immunostaining of vessels do not uniformly detect vessels of different sizes and can vary in efficiency in pathological processes. Therefore, we decided to compare the utility of anti-Collagen IV and anti-Factor VIII antibodies as markers for blood vessels in BB from COPD versus normal subjects and investigate what the differences are in the vessel profiles that they stain. Based on our previous findings [1, 2], we would expect any results in this study to apply generally to smokers as well as COPD.

Permeability of mucosal vessels in asthma has been reported to be increased and to correlate with clinical deterioration [15]. Vascular endothelial growth factor (VEGF) increases vascular permeability to blood water along with proteins [16]. We have reported increased vessel-related VEGF in the Rbm of COPD airways with the changes most marked for current smoking COPD patients [2]. Therefore, it is also reasonable to study vessel leakiness in the Rbm of bronchial wall in current smoking COPD subjects.

## 2. Material and Methods

This was a *cross-sectional study*. *Subjects *were recruited by advertisement. The study was approved by the *Human Research Ethics Committee (Tasmania) Network,* and all subjects provided written informed consent. Twenty-eight mild to moderate COPD subjects and eight normal nonsmoking controls participated. COPD was diagnosed using GOLD criteria [17]. Subjects with other respiratory diseases including a clinical history of asthma were excluded. All COPD subjects were on short-acting anticholinergic bronchodilators only. *Lung function tests* were performed according to ATS/ERS guidelines [18]. *Fiberoptic bronchoscopies and endobronchial biopsies* were performed as previously described [2]. There were no complications from the procedures.

### 2.1. Tissue Processing

**Figure 1:**
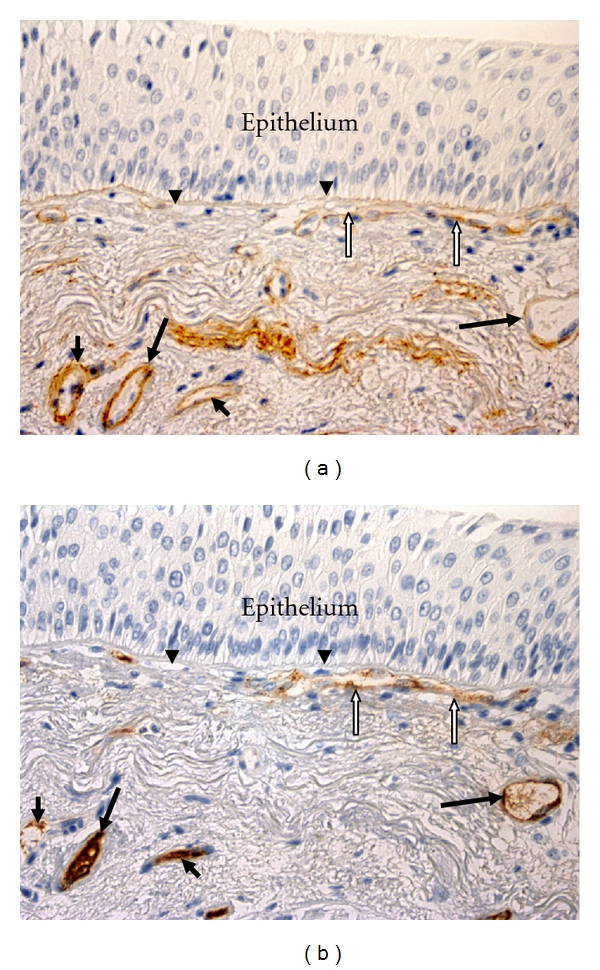
Vessels in the bronchial mucosa. Bronchial biopsies taken from the same current-smoking COPD subject, ×400. Vessels (black arrows) are stained with anti-Collagen IV antibody (a) and anti-Factor VIII antibody (b). A large vessel which contacts the reticular basement membrane (Rbm, arrowheads) is indicated by white arrows. The epithelium is thickened, probably because of chronic smoking exposure.

2 × paraffin-embedded sections of 3 *μ*m and 50 *μ*m apart mounted on APTS-coated slides were used. Following dewaxing and hydration, sections were subjected to heat retrieval using Dako S1700 for 20 minutes (except Albumin which did not require epitope retrieval), and then endogenous peroxidase was quenched using 3% hydrogen peroxide for 15 minutes. Sections were incubated in primary antibodies for either Von Willebrand factor (Factor VIII-related antigen) (Dako M06160), Type IV collagen (Dako M0785) (both at 1 : 150 for 90 minutes) (Figure 1), or Albumin (Abcam Ab 2406) 1/6000 for 30 minutes at 20 degrees Celsius. For negative controls, matched sequential sections were stained with primary antibody, replaced with a species-appropriate IgG1 isotype, at equivalent dilutions and conditions. A horseradish peroxidase (HRP) conjugated DAKO Envision plus (Dako K4001) reagent was used for secondary antibody binding and DAB PLUS (DAKO K3468) for color resolution (brown). Mayer's haematoxylin counterstain was used to elaborate nuclei. Sections were dehydrated in ethanol, cleared in xylene, and mounted in Permount prior to analysis.

*Measurements* were performed using a computer-assisted image analysis tool (Image-Pro version 5.1, Media Cybernetics, USA). Pictures of all intact and nonoverlapping areas were taken from each slide, and eight separate fields were chosen randomly for measurements to have on average 3 mm of the Rbm in our measurements as we did in our previous report [2]. The histologist (AS) who performed the measurements was blinded to the diagnoses and order of slides, which had been independently randomly sorted and coded.

To be constant, we used the same methods for measurements so as to be able to compare the results of this current study with our previous report [2]. Vessels in the reticular basement membrane (Rbm) and down to a depth of 150 *μ*m of the subepithelial lamina propria (LP) from the antilumenal margin of the Rbm were measured separately. Only well-formed cylindrical or tubular structures that were stained with immunostaining antibodies were measured as vessels to avoid including nonvascular cells in analyses (Figure 1). Number and cross-sectional area of vessels were measured. These data were normalized by dividing by the length of the Rbm or dividing by the surface area of the LP examined. Mean vascular size (MVS) was calculated as total vascular area/number of vessels. The area of the LP excluded mucous glands and muscle.

For vessel permeability, using Image-Pro 5.1 again, the percentage of compartment tissue area stained for albumin was measured separately in the Rbm and LP in current smoking COPD and normal controls and the results expressed as a percentage (*μ*m² of tissue stained/*μ*m² tissue examined ×100). Finally, the percentage area of perivascular albumin staining was measured within the 10 *μ*m perimeter around vessels (to avoid overlapping of areas) in both the Rbm and LP. We have previously successfully used this method to measure vascular permeability in asthmatic airways [15].

### 2.2. Statistical Analyses

The data from the two methods of vessel staining were tested for agreement using the method reported by Bland and Altman [19]. Very briefly, by this latter method, the means of the two measurements are plotted against the differences between the two measurements. The 95% limits of agreement (LoA = mean of differences ±2 standard deviations (SD) of differences) were calculated for every measurement.

For comparison of means between two groups or between two methods of staining, Student's *t*-test was used for variables with normal distribution and the Mann-Whitney test for nonnormally distributed variables. Fisher's Exact test was used to compare gender distribution between two groups. All continuous data were presented as median (interquartile range), except for the data that are included in agreement between the two methods of vessel staining which were presented as mean ±2 standard deviations. *P* value less than 0.05 was considered as significant. SPSS 16.0 was used for statistical analyses. Pearson's or Spearman tests were used to test correlations for normally and nonnormally distributed variables, respectively. SPSS 16.0 was used for statistical analyses.

## 3. Results

Thirty-six subjects participated in the study.  Table 1 summarizes the demographics. The 28 COPD subjects were balanced between 15 current smokers and 13 ex-smokers. There were no significant differences between groups in age or gender.

### 3.1. Collagen IV versus Factor VIII

**Figure 2:**
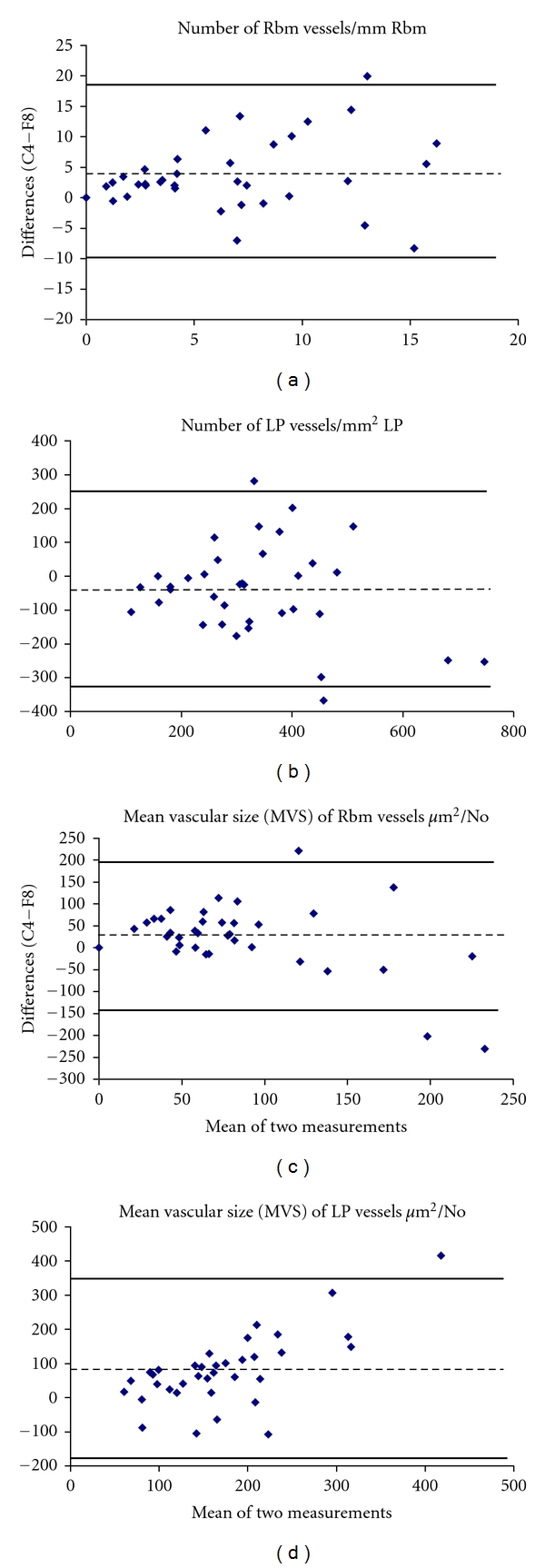
Bland and Altman plots for number of vessels and mean vascular size (MVS) in the reticular basement membrane (Rbm) and lamina propria (LP) in all study subjects together. Bland and Altman plots illustrate agreement between the two methods of vessel staining with anti-Factor VIII and anti-Collagen IV antibodies. Values on the *X* axis represent means of the two measurements; values on the *Y* axis indicate measurements with Collagen IV staining minus measurements with Factor VIII staining (C4-F8). The broken line represents the mean difference. Bold lines indicate Limits of agreements (mean of differences ±2 standard deviations). C4: anti-Collagen IV; F8: anti-Factor VIII.

**Figure 3:**
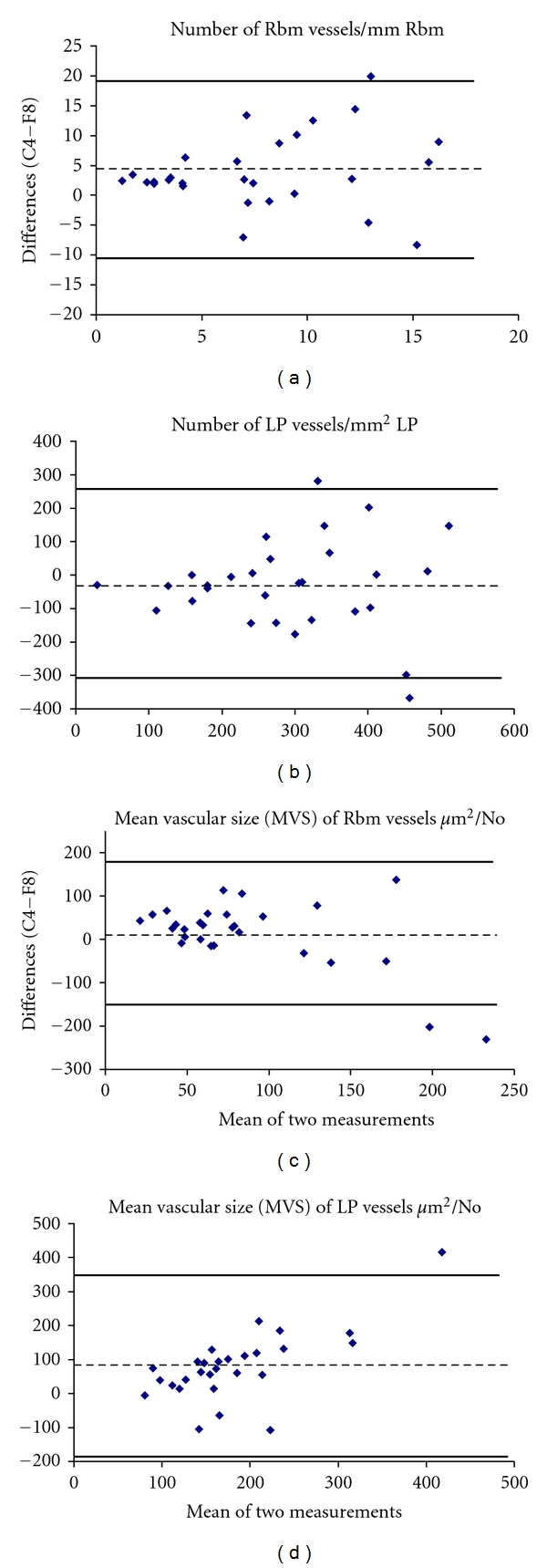
Bland and Altman plots for number of vessels and mean vascular size (MVS) in the reticular basement membrane (Rbm) and lamina propria (LP) in COPD subjects. Number of vessels is expressed as No/mm Rbm and No/mm² LP. MVS is presented as area *μ*m²/No. For MVS in the LP, the agreement between the two methods was best when vessels were relatively smaller and the differences increased as MVS increased, with anti-Collagen IV showing larger MVS than anti-Factor VIII. Description of the measurements and abbreviations are presented in Figure 2.

The most important characteristics of agreement between the two methods of vessel staining are summarized in Table 2. Bland and Altman plots show that anti-Collagen IV antibody detected higher number of vessels and larger MVS in the Rbm and lower number of vessels but again larger MVS in the LP compared with anti-Factor VIII antibody in all study groups (Figure 2). This was also the case for the COPD group analyzed separately (Figure 3). For Bland and Altman plots, values on the *Y* axis in Figures 2 and 3 were calculated as measurements with Collagen IV staining minus measurements with Factor VIII staining.

**Figure 4:**
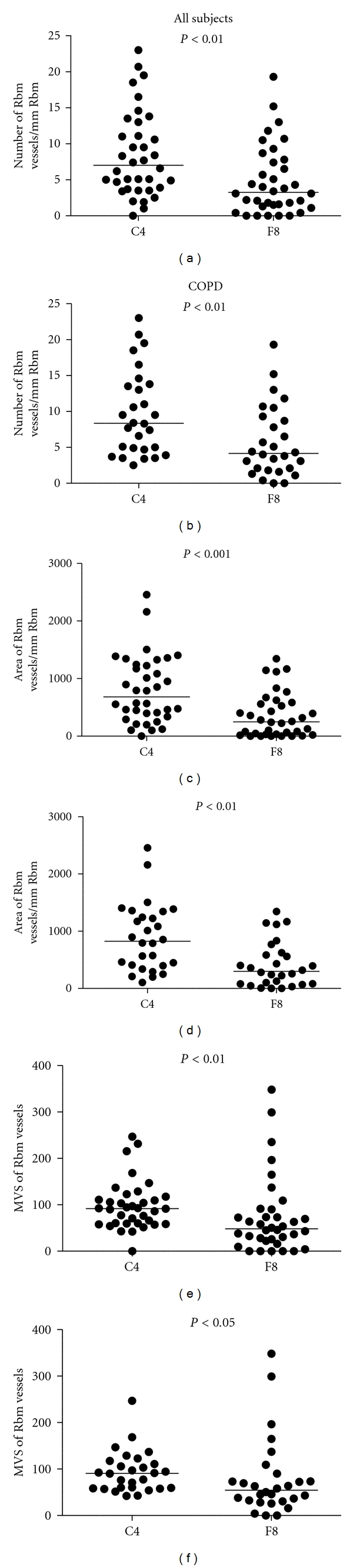
Comparison of the number, area, and MVS of vessels in the reticular basement membrane (Rbm). In both COPD and normal groups, Collagen IV staining outlines more vessels of larger caliber, than Factor VIII staining. MVS: mean vascular size (area *μ*m²/No); C4: anti-Collagen IV antibody; F8: anti-Factor VIII antibody.

**Figure 5:**
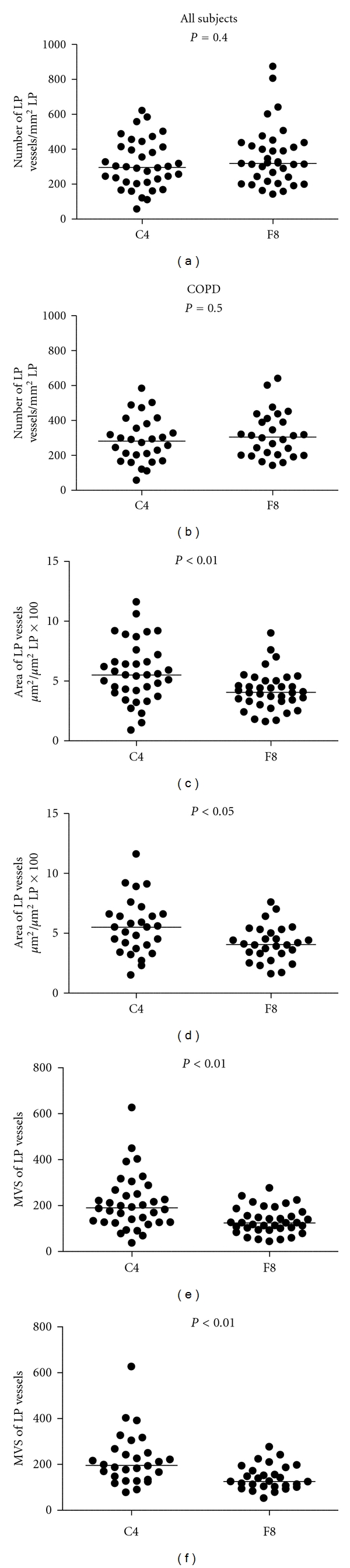
Comparison of the number, area, and MVS of vessels in the lamina propria (LP). In both COPD and normal groups, Collagen IV staining outlines bigger vessels but not more vessels. MVS: mean vascular size (area *μ*m²/No); C4: anti-Collagen IV antibody; F8: anti-Factor VIII antibody.

Comparing the means of the absolute number, area and MVS of Rbm vessels stained by the two immunostaining methods confirmed our results with the Bland and Altman plots; there were significantly greater number, area, and MVS of vessels with anti-Collagen IV antibody than with anti-Factor VIII antibody both when all subjects were tested together and when the COPD group was tested alone (Figure 4). In the LP, comparison of the means showed that the differences between two methods of blood vessel staining were significant for area and MVS but not for the number of vessels (Figure 5). 

### 3.2. COPD versus Normal Controls

**Figure 6:**
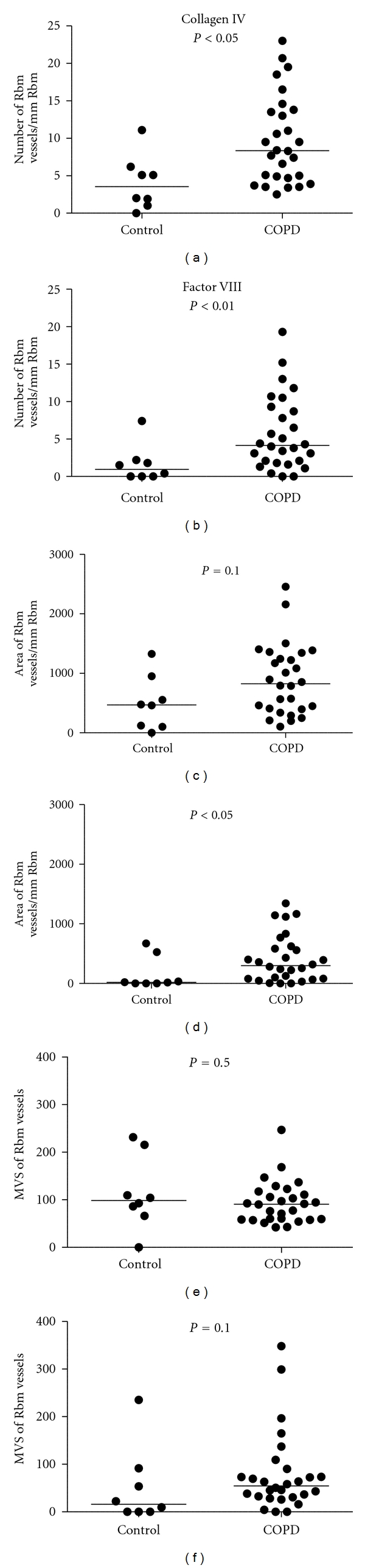
Comparison of reticular basement membrane (Rbm) vessels in COPD and normal control groups. COPD subjects have more vessels with both methods of staining. MVS: mean vascular size (area *μ*m²/No).

**Figure 7:**
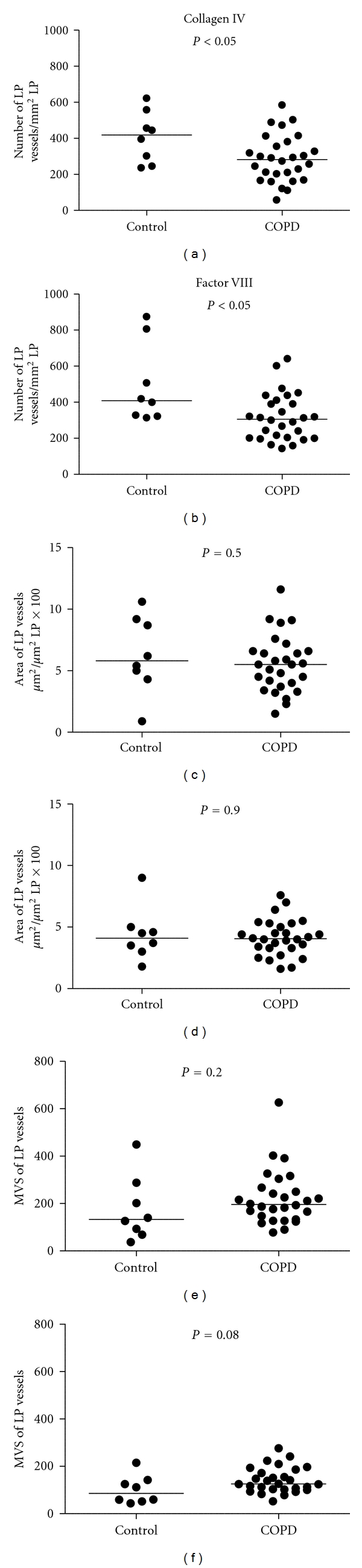
Comparison of lamina propria (LP) vessels in COPD and normal controls. Vessel number is decreased in COPD with both methods. MVS: mean vascular size (area *μ*m²/No).

 When COPD subjects were compared to controls, COPD had significantly more vessels in the Rbm and fewer vessels in the LP with both Collagen IV and Factor VIII antibody staining (Figures 6 and 7). 

Significantly, more tissue was stained for albumin in the Rbm in current smoker COPD than controls (median (interquartile range), *μ*m²/*μ*m² presented as percent, 0.37% (1.68) versus 0.00% (0.20), *P* = 0.02). But perivascular albumin in the Rbm or LP and albumin staining in the LP were not significantly different between two groups. 

### 3.3. Correlations

Number of Rbm vessels stained with anti-Factor VIII antibody correlated negatively with forced vital capacity (FVC) only in ex-smokers with COPD (Spearman *r* = −0.8, *P* = 0.002). Otherwise, there were no correlations between our anatomical findings and lung function parameters. We did not find any suggestion of a relationship between either age or pack-years smoking and vascular or permeability changes in the COPD group.

## 4. Discussion 

This study showed that anti-Collagen IV antibody tends to stain more vessels in the Rbm and bigger vessels overall in both the Rbm and LP, while anti-Factor VIII antibody stains relatively smaller vessels. It has previously been shown that vessel markers can have different sensitivity and specificity in detecting vessels in normal versus abnormal conditions. For example, factors such as genetic diversity in endothelial cells, hypoxemia, age, and shearing stresses have effects on the expression of Factor VIII protein [10, 20, 21]. The sensitivity and specificity of these markers are also related to the size of vessels [9, 22]. Indeed, our data are consistent with a previous report showing that staining for Factor VIII cannot detect larger-sized vessels as accurately as smaller ones in invasive breast cancer [9]. But this type of differentiation has not previously been attempted with airway wall samples; although our previous work did show that there was a shift to greater number and smaller vessels in the LP in asthma [14]. 

Anti-Collagen IV antibody consistently detected larger vessels in the Rbm than Factor VIII antibody. In the LP, the agreement between the two methods was best when vessels were relatively smaller, so that as the MVS increased, there were increasing differences between the two methods, with anti-Collagen IV again demonstrating larger MVS than anti-Factor VIII (Figures 2 and 3). The literature would suggest that smaller vessels are likely to be disproportionately newer vessels, while larger vessels are likely to be older and more mature or even “post-mature” ghost vessels [23–25]. An in vivo study on mice showed that new vessels in airways, formed under angiogenic stimulation by VEGF, had detectable pericytes and basement membrane by day 7. When the newly formed vessels were deprived of VEGF, firstly the flow of blood stopped, followed by death and then fragmentation of endothelial cells, and finally apoptosis of pericytes. However, a basement membrane sleeve from the whole structure remained for some time [23]. This emphasizes that different markers will demonstrate vessels better or worse depending on their age, maturity, and growth factor environment. We would propose that in conditions where we expect active new vessel formation, such as active asthma or malignancies, anti-Factor VIII antibody may detect these newer vessels better than anti-Collagen IV antibody. This could also be potentially useful in evaluating the effects of treatments on vascular regression, for example, the effects of inhaled corticosteroids on vessels in the airways [13]. In contrast, vessels which are larger and probably older are more effectively detected when stained with anti-Collagen IV antibody rather than with anti-Factor VIII antibody. On this basis, however, we cannot explain why the number of Rbm vessels is significantly increased with anti-Collagen IV compared to anti-Factor VIII antibody; we rather expected the data to be the other way round, that is, more new and younger vessels. We suggest that this could be the result of a high number of aged vessels with well-formed endothelial basement membrane where the endothelium has sufficiently matured to lose its Factor VIII antigens. 

A novel and especially interesting finding of this study was increased leakiness of the vessels in the Rbm in current smoking COPD. However, in contrast to our hypothesis, we did not find a correlation between albumin staining in the Rbm and vessel-associated VEGF (data obtained in our previous study [2]). Leak of plasma and its protein material could provide an appropriate environment for angiogenesis by extravasation of fibrinogen and formation of a fibrin gel. Endothelial cells and other mesenchymal cells can easily settle and grow in this environment [26]. Therefore, this finding is compatible with the hypervascularity of the Rbm we have demonstrated in COPD. The consequence of having leaky vessels just below the epithelium in COPD is ripe for speculation and further study, but, at the very least, it may contribute to fluid and protein flux into the airway lumen and add to the mucus volume and constituents. This has never previously factored into the concepts of the pathogenesis of smoking-related airway disease pathophysiology. 

Both Collagen IV and Factor VIII immunostaining of bronchial biopsies in this study confirmed our previous findings of hypervascularity of the Rbm and hypovascularity of the LP in the COPD group compared to the control group [2]. The negative correlation between FVC (likely to reflect predominantly small airway narrowing) and Rbm vessels emphasizes the potential functionally detrimental effect of vascular remodeling in the bronchial mucosa in COPD. 

## 5. Conclusions 

 Larger and probably more mature vessels were detected better by anti-Collagen IV antibody, while smaller and probably newer vessels were detected better by anti-Factor VIII antibody. Increased permeability of vessels in the Rbm of current smoking COPD subjects could be related to the hypervascularity of this compartment and add to its potential functional significance. Both anti-Factor VIII and anti-Collagen IV antibodies confirmed hypervascularity of the Rbm and hypovascularity of the LP in the COPD bronchial mucosa compared to normal. 

## Figures and Tables

**Table 1:** Demographics.

	Group	
	Control (*n* = 8)	COPD (*n* = 28)
Age*, median (interquartile range), years	54 (9)	61 (10)
Gender^†^, female (number)	2	9
Pack-year smoking history, median (interquartile range)	0	47 (23)
FEV1/FVC%^¶^, median (interquartile range)	79 (17)	57 (16)

* *P* = 0.1 (Mann-Whitney test).

^†^ *P* = 0.2 (Fisher's Exact test).

**^¶^** *P* < 0.001.

**Table 2:** Agreement between two methods of vessel staining in both groups together.

	Mean, Collagen IV	Mean, Factor VIII	Mean of differences*	95% LoA^†^
Number of vessels/mm Rbm	8.5	4.8	+3.7	−10.2, +17.6
Area of vessels/mm Rbm	803	357	+446	−995, +1887
MVS of Rbm vessels, *μ*m²/number	98	73	+25	−143, +193
Number of vessels/mm² LP	313	356	−43	−333, +247
Area of vessels *μ*m²/*μ*m² of LP × 100	5.7	4.2	+1.5	−4.4, +7.4
MVS of LP vessels, *μ*m²/number	212	133	+79	−184, +342

*Calculated as measurements with Collagen IV minus measurements with Factor VIII antibody.

^†^LoA: limits of agreement (mean ±2 standard deviation).
